# Comparing Apples to Oranges: Comparative Case Study of 2 Produce Carts in Chicago

**DOI:** 10.5888/pcd11.140170

**Published:** 2014-08-14

**Authors:** Katherine Wright, Lauren Anderson

**Affiliations:** Author Affiliation: Lauren Anderson, Northwestern University, Chicago, Illinois.

**Figure:**
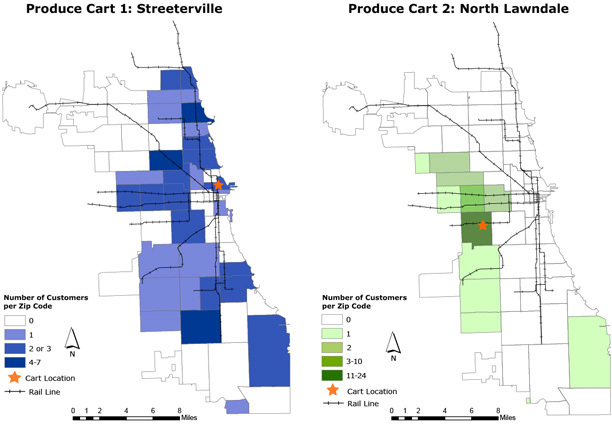
**Maps**. Customer utilization of produce carts, Chicago, 2012. These maps show the service area of 2 mobile produce carts launched during the summer of 2012 in 2 Chicago neighborhoods. Survey data recording self-reported customer demographics were collected for each produce cart to visualize customer reach as part of program effectiveness. Cart 1 is outside an underserved zone in the centrally located Streeterville neighborhood. Cart 2 is on the west side of the city in an underserved zone in the North Lawndale neighborhood.

## Background

In June 2012, the Chicago Department of Public Health passed ordinances to legalize mobile produce vending throughout the city, provided at least 50% of produce carts operate in designated underserved areas. In response, the Neighbor Carts program emerged to promote the opportunity for economic success and healthful food access through an unconventional retail structure. Neighbor Carts are independent produce carts selling fresh fruit, vegetables, and nuts. Part food access enterprise and part workforce initiative, this program employs people who have previously experienced homelessness, addiction, or other barriers to employment.

## Methods

An evaluation was conducted through use of survey, interview, observational, and geographic information systems (GIS) data to define and visualize program effectiveness. The Northwestern University institutional review board reviewed and approved the project. Data for these maps were generated via a customer intercept survey that was conducted immediately after a purchase was made (n = 98). Twelve customer surveys were from residents outside of Chicago, from mostly suburban areas, and are not included on these maps. Questions focused on dietary habits, suggestions for program improvement, overall purchase satisfaction, and geo-demographic information. Carts typically operate Monday through Friday from 7 AM to 4 PM, weather permitting, from April through November. Customers from 6 carts were surveyed as part of the larger evaluation (1). These maps highlight customer zip code data from 2 carts to visualize differences in each service area.

## Main Findings

Cart 1 is outside an underserved zone (as defined by city ordinances), in a centrally located commercial hub just a few blocks west of major shopping and tourist attractions. The map for Cart 1 shows that it reached customers from many areas of Chicago, including those from underserved zones on the south and west sides of the city. Cart 2, in an underserved zone, experienced much higher use from customers living in closer proximity to the cart. Results from the larger evaluation demonstrated additional differences between the 2 carts, showing variation in customer age, sex, employment status, and Supplemental Nutrition Assistance Program (SNAP) participation (L. M. Anderson, MEd, unpublished data, April 2014). Both carts had a broader service area than a typical 0.5-mile walking radius, as was originally anticipated.

## Action

In an effort to prevent chronic diseases associated with poor nutritional intake and increase healthful food access, mobile produce vending programs have been identified as a promising strategy. These maps add to the growing body of evidence that the food environment is highly complex. Carts close in proximity but in different neighborhoods demonstrated unique consumer trends. Future evaluation must encompass a more nuanced look at program effectiveness, taking place on a microlevel to take neighborhood context into account. Factors including community engagement, proximity to public transportation lines, and local business support shape the food landscape and contribute to cart success in varying degrees, depending on the neighborhood. Additionally, it may be necessary to incorporate different evaluation metrics to determine cart success. Although sales volume is a key metric for individual cart sustainability, customer volume should also be considered. More specifically, if carts reach a broad service area that goes beyond a walking radius to provide access to fruits and vegetables to residents of underserved zones, then one of the over-arching program goals, to increase access to fresh produce to all Chicagoans, is accomplished.
